# Exploring the value of qualitative research films in clinical education

**DOI:** 10.1186/s12909-015-0491-2

**Published:** 2015-11-27

**Authors:** Fran Toye, Sue Jenkins, Kate Seers, Karen Barker

**Affiliations:** 1 Nuffield Orthopaedic Centre, Oxford University Hospitals NHS Foundation Trust, Windmill Road, Oxford, OX3 7HE UK; 2 Department of Anaesthetics, Intensive Care and Pain Medicine, School of Medicine, Cardiff University, Cardiff, UK; 3 Royal College of Nursing Research institute, Warwick Medical School, University of Warwick, Coventry, UK; 4 Nuffield Department of Orthopaedics, Rheumatology and Musculoskeletal Sciences, University of Oxford, Oxford, UK

**Keywords:** Qualitative research, Education, Chronic pain, Professional-patient relations, Personal narratives

## Abstract

**Background:**

Many healthcare professionals use both quantitative and qualitative research to inform their practice. The usual way to access research findings is through peer-reviewed publications. This study aimed to understand the impact on healthcare professionals of watching and discussing a short research based film. The film, ‘Struggling to be me’ portrays findings from a qualitative synthesis exploring people’s experiences of chronic pain, and was delivered as part of an inter-professional postgraduate e-learning module. The innovation of our study is to be the first to explore the impact of qualitative research portrayed through the medium of film in clinical education.

**Methods:**

All nineteen healthcare professionals enrolled on the course in December 2013 took part in on-line interviews or focus groups. We recorded and transcribed the interviews verbatim and used the methods of Grounded Theory to analyse the interview transcripts.

**Results:**

Watching and discussing the film became a stimulus for learning : (a) *A glimpse beneath the surface* explored a pro-active way of seeing the person behind the pain (b) *Pitfalls of the Medical Model* recognised the challenge, for both patient and clinician, of ‘sitting with’ rather than ‘fixing’ an ill person; (c) *Feeling bombarded by despair* acknowledged the intense emotions that the clinicians brings to the clinical encounter; (d) *Reconstructing the clinical encounter as a shared journey* reconstructed the time-constrained clinical encounter as a single step on a shared journey towards healing, rather than fixing.

**Conclusions:**

Films portraying qualitative research findings can stimulate a pro-active and dialectic form of knowing. Research-based qualitative films can make qualitative findings accessible and can be a useful resource in clinical training. Our research presents, for the first time, specific learning themes for clinical education.

## Background

How can qualitative research findings be used in clinical education? One of the difficulties of evaluating the impact of qualitative research is that findings provide an *interpretation*, rather than neatly packaged facts. This creates a danger that qualitative findings are side-lined for more tangible forms of ‘factual’ knowledge. We aimed to explore the impact of a short film portraying qualitative research findings on healthcare professionals who watched it. Appraisal of research impact tends to focus on measurable outcomes [1, 2]. Our construction of impact resonates with Parsons and colleagues; ‘impact might be a subtle shift in viewers’ perspectives’ which may usefully inform therapeutic encounters. Parsons and colleagues highlight the issue – what is the nature of impact in qualitative research? In their evaluation of an arts installation to portray the experience of homelessness, Parsons, Hues & Moravac explore how the audience interacts with research findings portrayed in art [1]. Similarly, we aimed to explore how viewers constructed meaning from the film, rather than to identify specific outcomes.

Our study is underpinned by a constructivist philosophy of knowing the world [3]. Although there are diverse ways of *knowing* [4], evidence-based medicine has a strong strand of objective modes of knowledge, or *Episteme*. Greenhalgh invites us to challenge accepted ways of *knowing*, by asking ‘what is this knowledge we seek to exchange?’ [5]. Other relevant forms of knowledge include *Phronesis* - an intuitive, tacit or practical wisdom [6]. It may be more useful to consider knowing as a *dialectic,* rather than *linear* process [5, 7]. Central to dialectic theory is the idea that tension between different ideas can create innovative ways of thinking [8]. This is in line with ‘situated judgement’ [7] or *tacit* knowledge [9] and resonates with anthropological texts that support the re-enactment of knowledge [10]. Within this dialectic framework, *knowing* can be conceptualised as a dynamic process that occurs at the *interface* between research findings and audience.

The use of artistic media (such as film or drama) to present findings, lends itself to an interactive, or dialectic, style of learning [11–19]. P*erforming* qualitative findings, aims to *evoke*, *provoke* and *stimulate* ideas [13], rather than present facts, and can be powerful because it facilitates emotional engagement beyond that from reading reports [1, 2]. Performative methods have been used in clinical education to facilitate learning through dialogue [2, 20–22], and to develop empathetic understanding [15, 16, 23, 24]. Through film, viewers can access different perspectives in a safe environment and explore their own clinical practice. Existing reviews suggest that there is a paucity of research exploring film as a dissemination mode [25, 26–27].

### Aim

We aimed to understand the impact on healthcare professionals of watching and discussing a short film that portrays the findings from a qualitative systematic review of patients’ experience of chronic musculoskeletal pain. Specifically, to explore how viewers constructed meaning from the film.

### Originating research and film

**Fig. 1:**
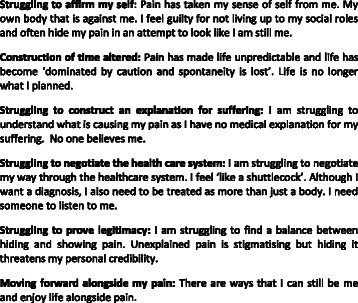
Summary of themes from the qualitative systematic review. This gives a summary of findings from Toye and colleagues qualitative systematic review of patients’ experience of chronic musculoskeletal pain [28, 29]

The film ‘Struggling to be me’^1^ is available on YouTube and presents findings from a qualitative systematic review of more than one thousand adults’ experiences of chronic musculoskeletal pain [28]. Studies in the original systematic review included a range of countries (Iceland, Northern Ireland, Switzerland, Finland, Netherlands, New Zealand, Australia, Canada, Norway, USA, Sweden and UK). The themes from this review [28, 29] are summarised in Fig. 1. Narrative exemplars for each theme were crafted together to form a script and an actor was chosen, following an audition, to tell ‘Sarah’s’ story of what it is like to live with chronic pain. The film was produced in collaboration with a professional visual media agency and was funded by the National Institute of Health Research (NIHR), UK, as an output from the originating systematic review [28]. The idea to explore healthcare professionals’ experience of watching the film came from two personal experiences where FT and KS were presenting the review findings, alongside the film, to audiences of healthcare professionals. On two occasions the film evoked an emotional response that the oral presentation did not evoke: ‘I don’t know why Fran, but I got really annoyed and cross with the woman in the film, and I wasn’t cross when I heard *you* read out her words’. Following its launch on YouTube, this film was utilised as part of a pain management course for health professionals.

## Methods

### Ethics

We obtained ethical approval from The Medical School Research Ethics Committee (MSREC), School of Medicine, Cardiff University. Participants were sent written information about the study, and offered an opportunity to discuss the study with SJ or FT. They then signed a consent form, which was returned by post. This was signed by SJ and a scanned pdf final version was emailed back to the participant. In addition at the beginning of each focus group video call, SJ asked if everyone was happy for the video call to be recorded and for their contribution to be used for the study.

### Sample

Nineteen qualified healthcare professionals undertaking an inter-professional postgraduate, level 7 (MSc level), e-learning module were recruited into the study. The sample was predominantly General Practitioners (eleven) and also included three nurses, three pharmacists, one physiotherapist and one psychiatrist. All worked in the UK, mostly within the Primary Care setting, or in partnership with it. All had an interest in chronic pain management. The film was delivered as part of the evidence based 'Bio-psychosocial' module. As the group included a single physiotherapist and psychiatrist, we do not identify participants by professional grouping to maintain participants’ anonymity.

#### Data collection

Four focus group interviews, and two individual interviews with participants who were unable to attend a focus group, took place on ‘Oovoo’ (a video chat application that allows you to make verbatim recordings). It could be argued that there are methodological limitations for interviewing participants on-line that go beyond the technical considerations. This online method, although allowing us to access diverse geographical locations throughout the UK (including Scotland and Northern Ireland), was likely to have had an effect on the performative aspects of the interaction. For example, participants were instructed to indicate, non-verbally, if they want to contribute to a particular line of discussion and not to interrupt or talk at the same time as someone else. In addition to this, body language was obscured because the screen only showed participants faces. However, despite the possible disadvantages, we wanted to explore the film’s impact in a specific educational setting where it was being used as part of e-learning Masters Module. As the film was a compulsory part of the module and its assessment this allowed a unique opportunity to explore its impact in clinical education. FT observed that this online method seemed to downplay potential power-play between professional groups. For example, it was very difficult for her to identify specific professionals, whereas this might have become much clearer in a face-to-face meeting. This may also have been because participants were enrolled together on the same educational course. We used a combination of focus group and individual interviews as two participants were unable to attend online groups. Although we recognise that group and individual methods will each create a different form of data, for the purposes of analysis we treated each transcript the same, focusing on the content of each transcript. Although we identified similar themes across data collection methods, a limitation may be that we have not fully considered the influence of context.

**Fig. 2:**
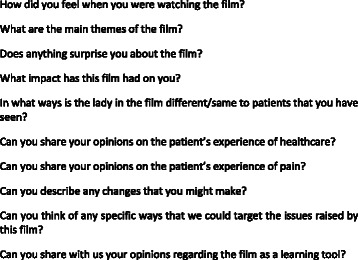
Sample interview questions. This provides a sample of questions used in the focus group interviews

Participants were invited to watch the film in their own time, and to consider some questions before attending the groups (Fig. 2). Groups took place at times convenient to the participants (weekend/evenings) and were facilitated by SJ. FT or KB attended to record observations, and to assist in the event of technical hitches. Interview time was limited to approximately one hour so as not to encroach too much on personal time. Recordings were transcribed verbatim and loaded onto Nvivo 9 software for qualitative analysis.

#### Analysis

This study was set within a constructivist Grounded Theory framework [30], taking the stance that knowledge is not ‘discovered’ but co-constructed. We aimed to explore the construction of knowledge when healthcare professionals watched the film, and therefore our study is strongly influenced by narrative thinkers, such as Frank [6, 31]. However, our analytic focus is on thematically coding interview *content* with the aim of developing a conceptual model that describes the co-construction of meaning between viewers and the film. We recognise that attending primarily to content can obscure the researchers role in data co-construction, and we therefore made every effort to combine our coding with ‘close analysis’ of each case: For example, by utilising extended exemplars, attending to the motives and silences of each speaker and playing attention to the details of telling and to choices of words. We also recognise that as researchers we played a significant role in the construction of the narrative, and made every effort to create a space where participants felt free (and safe) to discuss areas of learning from the film.

Thematic coding involves an iterative process of constantly comparing data, codes and categories within and across cases, and moving from an initial tentative category towards progressively abstracted theoretical categories that are grounded in the data. Our approach to research quality, outlined by Toye and colleagues in a recent BMC publication [32], strongly resonates with Eakins and Mykhalovskiy’s concept of ‘substantive judgement’ [33]. Thus whilst we report aspects of method to serve as a ‘positioning device’ [33] that allow the reader to understand our analytic context, our primary focus was the rigour of our conceptual analysis: (a) we used constant comparative methods (b) we collaboratively challenged analytic decisions during the coding process, and (c) use extended narrative exemplars that allow the reader to judge our analytical decision making.

## Results

We identified four related conceptual categories: (a) a glimpse beneath the mask; (b) pitfalls of the medical model; (c) bombarded by despair; (d) reconstructing the clinical encounter as a shared journey. We use interview excerpts to illustrate these categories and refer to the woman in the film as ‘Sarah’. We have used pseudonyms for participants to demonstrate that we compared themes across dataset, whilst retaining participant anonymity.

### A glimpse beneath the surface

This theme describes a growing understanding of the patient as a fellow human-being facing profound personal loss. In this way the film humanises the patient. Participants vividly described the impact of pain on Sarah’s sense of self. In the following exemplar, Sonia no longer refers to the patient as *he* or *she,* ‘pain changes *you* into someone you weren’t before’. She describes the difficult struggle to negotiate the *wide bridge* between the present self and the self that you *would* have been or *should* be.

Sonia (individual interview): I think one of the main themes [in the film] for me is how experiencing chronic pain like this changes you into being somebody who you weren’t before . . . issues around your sense of self identity and the loss of your old self, the loss of . . . very important things in your life . . . . you imagine that you would be somebody and . . . have feelings of who you *ought* to be . . . and that difference can be very wide and it can be a bridge that's very, very difficult to, to cross.

There is a sense of emotional engagement and identification with Sarah, demonstrated through words like ‘powerlessness’ and ‘heaviness’. Josh talks about the ‘universal sentiment’ of suffering as experience that we can all understand and share; ‘haven’t we all had to think about our bodies at certain times?’

Josh (focus group 2). . . . A sense of loss of identity disconnected from herself, powerlessness that she felt and that sense of not knowing who she was anymore and the identity she'd forged . . . about who she was [now] slipped through fingers somehow . . . and it that was the heaviness I think . . . it felt very, very universal sentiment potentially . . . . Accentuated when it’s made part of your every living, breathing moments . . . . We’ve all had to think about our bodies at certain times you know so it's that identification in that regard I think. If that makes sense?

The film gave participants an unexpected *glimpse* beneath the surface. Some were surprised that they had not stopped to think about the profound effect of pain on their patients’ lives; they talked about being ‘struck’ or ‘surprised’ or that ‘I didn’t expect’.

Sheila (focus group 1): I was very struck how her pain seemed to really affect her identity as a person and I don't think that I had . . . I kind of had reflected on that in the past, but not as quite much as with that film.

For Grace, it struck a chord that Sarah was embarrassed by her pain:

Grace (focus group 4): I think I was just a bit surprised she was very embarrassed by her pain . . . [she was] quite ashamed I think. I suppose I never looked into that side of things . . . I didn't expect that from the patient it's just not something I'd thought in depth about before . . . I just thought . . . that struck a chord with me.

Deena described it as ‘frightening’ to realise that in the past she may have prejudged patients like Sarah, rather than truly *seeing* them. In previous clinical encounters she hints that she had focused on her own feelings about the kind of patient who made your ‘heart sink’ because they didn’t seem to get any better. There is a sense that things are different following the film, and that she now ‘understands’.

Deena (focus group 3) It was like getting a glimpse into many such patients I have seen . . . the most frightening thing was when she said, 'people can't really see this pain' . . . *and this is so true* because they come into your consulting room with a smiling face and, and you know, with make-up and everything and you tend to have this *prejudice* say, thinking ‘oh this is one of those heart sink patients’ . . . my outlook is changed . . . I felt really you know, good watching the video because it really helped me to you know, to understand these kind of patients better.

#### I can now see her struggle to perform pain

Participants discussed Sarah’s hidden struggle to strike a balance between hiding and showing her pain. Marion describes her insight into the performance of pain like wearing a mask or putting on your makeup.

Marion (focus group 4): Many patients they put on a mask especially for those around them because they don't want them to see how they're feeling and they don't want to focus on their pain on a daily basis. So it is kind of almost like putting your make-up on every morning.

Deena’s story hints that in the past she has erroneously judged the legitimacy of patients’ pain because of their outward ‘demeanour’. Her narrative demonstrates her exploring how healthcare professionals judge what pain *really* looks like. She strongly emphasises the value of the unexpected lesson not to judge a book by its cover, which has come as ‘a great surprise’.

Deena (focus group 3): The demeanour of a patient when they come in, like when she was in front of the [mirror] putting make-up on, and how she was trying to justify, ‘that’s just part of how we present ourselves to people’ - that doesn't mean she is not in pain. And that is so important I think to understand, because sometimes we discuss patients, we say ‘oh she came in with back pain but I don't think she's *really* in pain’ you know . . . but *really* even if somebody is in pain and distress, [it] doesn't always have to be in how they present themselves and I think that came as a huge surprise to me.

Despite this valuable lesson, Christopher interprets a mismatch between Sarah’s outward appearance and her description of pain and personal loss. This mismatch makes him doubt the authenticity of Sarah’s pain and threatens her personal credibility. Christopher’s story shows how he sees Sarah through the gaze of a clinician - ‘a cold clinical observation’- implying that as a clinician he can see what others might miss:

Christopher (focus group 2): If I could just make a cold, cold clinical observation, but there really was an incongruity between the symptoms and life that the lady portrayed verbally and what she was actually doing in the video. She was able to walk on the promenade she was able to go to the coffee shop, she was able to mobilise without apparent difficulty, travel on a bus, she was well presented and able to care for [herself] . . . . there is a little bit of a mismatch . . . the language and the behaviour were slightly out of sync.

However, he feels ‘uncomfortable’ because he cannot reconcile his clinical mistrust of her with his knowledge that appearances are deceptive. His discomfort prompts him to seek approval from the group by asking ‘does that make sense?

Christopher (group 2): [I was] trying to justify myself because I feel a bit uncomfortable I have to say in myself in saying that it seemed odd that she was loading the car, but it is the fluidity of how she loaded the car, does that make sense?

Other stories describing clinical mistrust of patients highlight a need for healthcare professionals to ask more ‘searching questions’ in order to truly reveal what lies beneath the surface of a patient’s outward appearance. Participants begin to question their implicit trust in the value of what the clinician *sees* above what the person *says*. For example, Clara admits ‘you wouldn’t know by *looking’* that they are in so much pain.

Clara (focus group 4): I've often been surprised if I try and get them to score their pain . . . often their scores are very different to what I think they're going to be especially in the ones that are quite [mmm] you know by *looking* at them you wouldn't think that they were in pain . . . .they're not showing that they’re in pain. So when they say that they've got an 8/10 score it often it still surprises me, so I think . . . to be asking more searching questions would perhaps give us a clearer idea of exactly [mmm] what their quality-of-life is and how much pain they're actually in.

Deena tells a story of a recent clinical encounter which has had a profound and ‘amazing’ effect on her by demonstrating that listening to patients and affirming their credibility can have a significant therapeutic role.

Deena (focus group 3): I think it [has] actually given a good sort of addition to my toolbox . . . because once you understand your patients, it’s so much easier . . . sometimes all they want is to be listened to . . . listen to what they have to say . . . what they want is empathy and understanding and to recognise their problem as a valid problem . . . it has really helped me . . . Recently you know, I was in the clinic and I actually had a patient coming, with long-standing pain and all I did was listen to her . . . she just got up and said that ‘this is the first time I really felt you know, *heard* and thank you and she said ‘I'm feeling already a better person’. I just thought that was amazing.

#### The challenge of clinical time constraints

Participants justify why imposed time constraints encourage healthcare professionals to focus on treating *the biological body*, by arguing that the biomedical approach i.e. *the system* facilitates a speedier consultation. There is also an underlying expectation that the primary ‘problem’ to be solved within these time constraints is diagnosis and treatment. There is a sense that it would be hard work for them to change this expected formula, ‘it’s far easier to reach for the prescription pad’.

Sonia (individual interview): I think as a clinician [you] focus straightaway on . . . a biological type approach to it. I think some of the psychological feelings get more brushed over perhaps . . . very often there is not the space in the consultation . . . we're immediately trying to deal with the problem; listen, deal with the problem, make a . . . printout prescription.

Graham (individual interview): The problem is, to discuss patients feelings and views and thoughts and so on is very very time consuming, now how do you organise that in general practice with 10 min consultation? . . It's far easier to reach for the prescription pad and say ‘try this’.

However, although participants justify that they only have time to focus on the physical body (not the person), there is an underlying tension; to be effective you actually need to invest more time to get to know the person. This dilemma remains unresolved, although Graham hints that time saved is a false economy.

Graham (individual interview): it’s certainly the way forward because, this type of investment [in time] is like an insurance policy you know, you pay into today to gain something tomorrow the more you invest in time and assessment today hopefully you will have a better results in the future and less chronicity, less healthcare costs, less consultations . . . we must find a way of concentrating this more.

### Pitfalls of the medical model

This theme describes how watching the film encouraged participants to explore the pitfalls of the biomedical model. Firstly, the challenge of the mind-body dualism, and secondly, the challenge of not being able to diagnose and *fix* a problem.

#### The challenge of breaking down the dichotomy of mind and body

Participants explored the challenges of treating chronic unexplained pain within a culture that hinges upon the dualism of mind and body. Graham hints that the biomedical model still predominates and that the film acts as a timely reminder of the ‘personal side’ of pain that medical science can neglect.

Graham (individual interview): This film has tended in a way to detach itself from the highly technical nature [of pain science] and concentrate on personal side of [pain experience] . . . a collaborative approach to patients in chronic pain . . . it is very important to remind ourselves that despite the technicalities involved is very much a personal aspect and it's very easily forgotten . . . with the evolution of biopsychosocial principles I think the biomedical side still predominates.

However, Cathy describes the stigma of the ‘psychological’ in our society that can make it challenging for healthcare professionals to adopt a biopsychosocial approach.

Cathy (focus group 3): I thought it was useful that she was actually verbalising the kind of stigma associated with having a psychological distress, I mean it's a lot more acceptable in our society to have some kind of physical problem, physical ailment.

Grace reinforces this notion of stigma by narrating how Sarah had ‘enjoyed’ the physical credibility of having a broken bone, as opposed to the discrediting status of medically unexplained pain.

Grace (focus group 4): Yeah when she described the broken arm you know she's in the car park and she describes the fact that she was so pleased she almost enjoyed having a broken arm because people could see that there was something wrong with her and she could justify you know how, how she was feeling.

Participants recognised that the stigma of having a psychological label could profoundly affect their patient’s personal credibility. Jenny confesses that as healthcare professionals we may be ‘guilty’ of adding to patients’ suffering by burdening them with this label. She recognises an inherent divide between clinicians and patients that can be bridged by ‘speaking in the right language’.

Jenny (focus group 1): The terminology . . . psychiatric and psychological . . . have a stigma attached to them that is not intended, and that was one of the things that I took from the video . . . we accept that patients with long term pain will have a *psychological component* to it but actually labelling it as that . . . can be quite negative . . . make sure that you are actually speaking in the right language to the patient . . . because I thought actually we are all guilty of it.

#### The challenge of ‘sitting with’ (not fixing)

Participants also explored the challenge of ‘sitting with’ a patient, as opposed to trying to diagnose and ‘fix’ the problem. Josh describes ‘uncomfortableness’ because he does not know how fix the problem. This discomfort is underpinned by a prevailing biomedical culture that expects a healthcare professional to diagnose, treat and cure. Josh recognising that it is very difficult for him just to *sit with* a person and not be able to help them:

Josh (focus group 2): What [the film] highlighted for me as a clinician is the *uncomfortableness* when you don't know what to do and how to stay with the patient despite that . . . and I suspect that was more about the clinician's uncomfortableness because it's very difficult to sit with someone when they’re pain and just listen and be there and not necessarily rush to fix.

Andrew wonders whether medical teaching reinforces this feeling of ‘uncomfortableness’. At medical school we are taught ‘assessment, diagnosis, treatment and improvement’; he hints that the challenge of treating patients with chronic pain may therefore reflect our own *vulnerabilities*. This has an implicit implication for education as it acknowledges a need to learn how to tell patients that we cannot ‘treat to improve’.

Andrew (focus group 3): It’s something that isn't taught . . . through medical school . . . the model really is assessment, diagnosis, treatment and improvement and I think doctors do find it difficult to acknowledge, it's not ingrained into us to tell a patient [that] we *can't* [treat] *. . .* to improve . . . . and I think our own inadequacies sometimes come through and actually a ‘heart sink’ patient is more a reflection of our own vulnerabilities and inadequacies in dealing with that.

Josh supports this feeling of vulnerability by asking whether clinicians sometimes choose to do diagnostic tests to fulfil a need to *do something* tangible. He advocates the need to be ‘very self-aware’ of our clinical decision-making; do we actually have ‘another agenda?’

Josh (focus group 2): [be] very self-aware when you're thinking about doing tests . . . I think sometimes in chronic pain doctors are doing the tests for themselves not for the patient. because again with that kind of, that uncomfortableness with not be able to fix or do something . . . sometimes questioning . . . what am I trying to achieve with this really . . . is there another agenda behind it? And I think that does happen it is lot.

### Feeling bombarded by despair

This describes how watching the film took participants ‘through all the emotions’ that Sarah was feeling. Kelly describes powerful feelings of ‘overall emptiness’ and ‘being ‘bombarded by despair’ whilst watching the film. With this emotional burden came empathy.

Kelly (focus group 3): Well, I mean there is an empathy . . . It was really not so much about the pain, so much, so much as there was an overall emptiness you felt a lot of empathy for [her] really . . . one wondered whether the pain had just taken the place of a social life, any meaning and purpose, any, any connection with anything really . . . it would be overall emptiness was what came through quite strongly and, and as I say very difficult to sort of work on that, because it was almost like being bombarded by despair.

#### It produced quite lot of intense emotions in me

Others recognised the intense emotional impact of watching the film. For example: feelings of depression, ‘quiet despair’ or futility. Kelly describes a ‘quite despair’ that resonates with narratives of ‘heart sink’ patients – just where do you begin?

Jenny (focus group 1): I actually felt really quite depressed myself by the time I had finished watching it . . . because it did actually take it you through all the emotions that she was experiencing . . . I felt quite depressed myself when I finished it.

Kelly (focus group 3): That quiet despair about where do you start with that lady . . . . yeah, despair is your first thing, where on earth do you start to begin?

Cathy suggested the potential use of the film in clinical education as it had allowed her to recognise the intense emotions that she might bring to this, and other, clinical encounters.

Cathy (group 3): I thought it was a really good educational tool . . . . Doctors are incredibly resistant to the thought they might be having an emotion about their patients. . . I would certainly find it very useful, very easily you get them to talk about their emotions, which they would normally attribute to the patient . . . that I thought, was excellent.

#### I’ve used the phrase detached empathy

Participants discussed the tension between the need to understand and empathise with patients and at the same time not becoming too personally involved. Christopher refers to this as a ‘detached’ or ‘professional’ empathy, hinting at an unspoken need for clinicians to remain at a distance for fear of harm to themselves - I must keep myself ‘*protected* professionally’ and not get too close.

Christopher (focus group 2): I think I had a very professional reaction to it . . . it was trying to listen to the person . . . sort of empathise . . . . [but] almost protected professionally . . . trying to see where that person was coming from but not letting it become too personal . . . I've used the phrase detached empathy.

#### It was a bit uncomfortable listening to the things she said

Participants also explored difficult feelings of wanting to defend the medical profession, and yet at the same time feeling frustrated and irritated by a profession that had let Sarah down. Sheila was surprised at her level of irritation when facing criticism from Sarah about her experience of healthcare; ‘you do want to defend your own’. This tension has a profound effect on Sheila who has thought about these feelings for ‘a long time’. She describes the personal challenge of negotiating divided loyalty

Sheila (focus group 1): I think that the only thing that surprised me was how irritated I felt when she said ‘I felt I was pushed from pillar to post’ . . . I really thought about that for a long time and thought ‘am I irritated because I want to be defensive of the healthcare profession and that we are genuinely trying to do our best? . . . yes the services aren't maybe as good as we would want them to be. . . [but] you do want to defend your own [laughs].

This sentiment is reinforced by Michelle who says that she finds it difficult to listen to Sarah’s ‘raw’ story. There is a mismatch between her underlying feelings that she is striving to do her best for her patient and what the film describes.

Michelle (focus group 2): I actually felt quite uncomfortable in, in some parts of it and I think that’s coming from the point of view of, you know, as clinicians we [are] striving and we like to think that we think we are doing the best for our patients, but when you hear it raw in that way and that's actually the way that patients are thinking, I find it, some bits quite difficult to listen to.

### Reconstructing the clinical encounter as a shared journey to healing

The final theme describes how participants made sense of what they had learnt from watching and discussing the film. Sense came from reconstructing the clinical encounter as a step on a shared journey towards healing, rather than focusing on an immediate biomedical fix.

#### We are going to chip away at this in bite-sized chunks

Participants discussed the values of seeing the clinical encounter as part of a collaborative process, a ‘journey’ taken in partnership with the patient, rather than as ‘my one chance’ to find a solution.

Michelle (focus group 2): See it as a journey rather than ‘this is my one chance at this and I have to get it all done in one go’ . . . if that's a sort of agenda that’s set with the patient from the start, then you both have a more realistic expectation of where things are going to take you.

Reconceptualising the clinic encounter in this way could actually take the tension out of time-constrained appointments. Josh describes how each clinical encounter can be constructed as another step forwards from a place of ‘stuckness’. His description moves us away from a place of ‘uncomfortableness’ at not being able to diagnose and fix, towards a collaborative process that takes place over time. Here the clinician is constructed as a facilitator, rather than director of healthcare. The healthcare professional role becomes one of recognising and congratulating small successes rather than fixing.

Josh (focus group 2): I think it's really useful having the 10 min in that regard because so much the time it's about moving away from that place of *stuckness* and creating a little bit of momentum . . . success sometimes is just getting out of bed and getting dressed you know and those little tiny bite sized chunks, recognising those as success and you can come back and say yes, that’s great you know you did that’, building on that momentum . . . you know it's the first bits of movement you know when you're pushing a car . . . [its] the first movement that is the hardest one, and then it builds.

#### This is a journey to healing not fixing

**Fig. 3:**
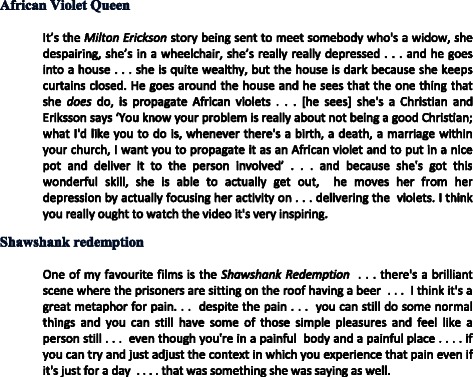
Metaphors used by participants to describe a transition from fixing to healing. This provides narrative examples of metaphors that participants used to describe an understanding of the transition from a focus on fixing the body to a process of healing

Participants explored how reconstructing the clinical encounter as part of a collaborative journey could facilitate a transition away from *fixing* towards *healing*. Participants used metaphors from popular culture to help them to describe and understand this transition (Fig. 3). Underlying these metaphors is the idea that a focus on enjoying the important things in life, rather than on curing pain, might open new possibilities for effective patient-clinician partnership.

Dahlia (focus group 4): Interestingly it wasn't as though she [Sarah] was hopeful that her pain would resolve or improve it was more the fact that she learnt that she would have to move on through her pain . . . focus on the things she enjoys . . . focus on the things that she would get more out of which I think were interesting elements which you could pass on to people as well.

Sonia encapsulates this focus on working alongside patients to achieve positive life increments in the term ‘sympathetic joy’. She describes the professionals’ role as one of generosity; recognising the patient’s personal struggle and congratulating achievements.

Sonia (individual interview): Recognise what strength the person has to be able to make that effort, that it could be an enormous effort to have to make . . . we should recognise that and congratulate them on doing what they can to try and help themselves. . . give a sense . . . that people have achieved something. Because I think everybody needs encouragement and we encourage and support all the time in, in life. But sometimes you know people continue to need encouragement and you have to have some sort of *sympathetic joy* with people who are obviously struggling, from the point of view of understanding where they're coming from.

#### Summary of findings – conceptual model

**Fig. 4:**
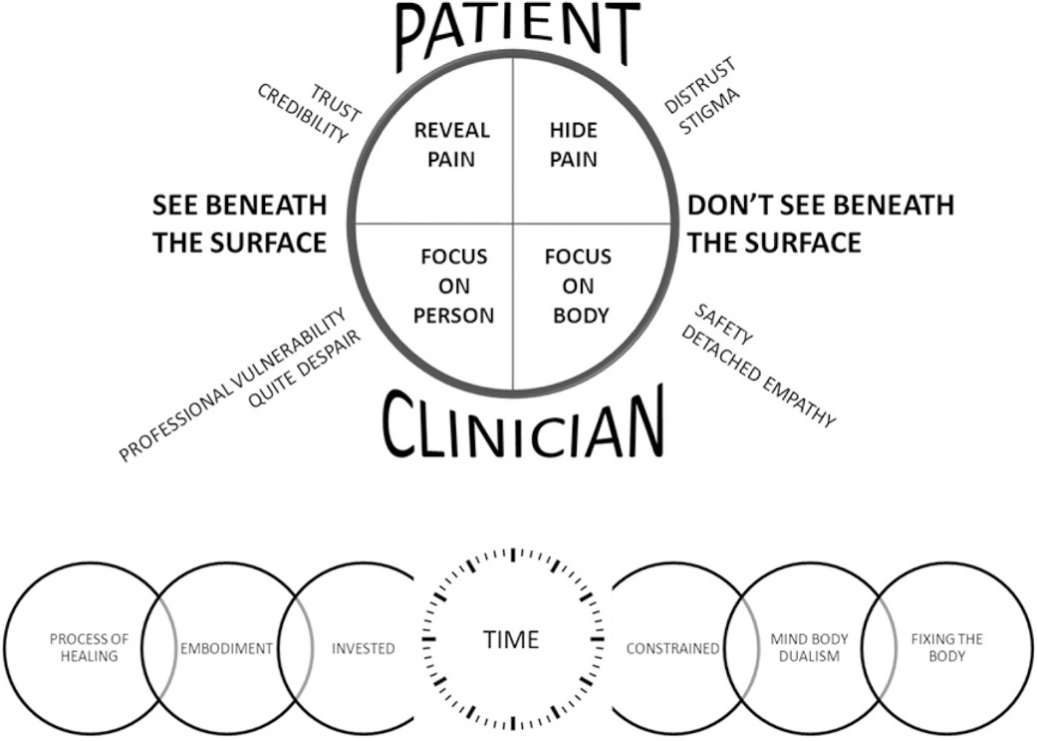
Conceptual model. This illustrates our conceptual model developed from the thematic findings. Central to that model is finding a balance between hiding or revealing pain and focusing on the physical body or the person. Hiding their pain, combined with focusing on the physical body can mean that the clinician does not see the person beneath their outward performance. Our findings demonstrate factors that might underpin clinicians decision to focus on the physical body or the person (professional vulnerability/safety/quite despair/detached empathy) and patients decisions to reveal or hide pain (trust/distrust, credibility/stigma). We illustrate how clinicians struggle to balance time constraints with a sense that it is necessary to invest more time to be clinically effective. Time constraints tend to favour a mind-body dualism and a focus on ‘fixing’ the body, whereas investing in time tends to supports an embodied approach to the clinical encounter with a focus on healing

Figure 4 presents a conceptual model that describes the co-construction of meaning between viewers and the film. Central to that model is (a) the patient struggling to find a balance between hiding and showing their pain and (b) the clinician struggling to find a balance between focusing on the physical body and also on the person. The model illustrates that the patient hiding their pain, combined with the clinician focusing on the physical body can mean that the clinician does not see the person beneath the outward performance. However, the decision to conceal pain is underpinned by the stigma of having medically unexplained pain and underlying feelings of distrust. The patients’ decision to reveal their pain, in combination with the clinician focusing on seeing the person, means that the clinician can begin to see beneath the surface. Although for the patient, a focus on seeing the person is underpinned by feelings of trust and personal credibility, it can lead to feelings of ‘quiet despair’ and professional ‘vulnerability’ for the clinician. Focusing on the physical body is described as a place of safety and ‘detached empathy’ (this is what I trained to do). Finally, clinicians struggle to balance time constraints with the sense that it is necessary to invest more time to be truly effective. Time constraints tend to favour a mind-body dualism and a focus on ‘fixing’ the body, whereas investing in time tends to supports an embodied approach to the clinical encounter with a focus on healing.

## Discussion

We aimed to understand the impact on healthcare professionals of watching and discussing a short film that portrays the findings from a qualitative systematic review of patients’ experience of chronic musculoskeletal pain. Specifically, to explore how viewers constructed meaning from the film. Our findings support the usefulness of films that portray qualitative research findings for clinical education. Watching and discussing the film ‘Struggling to be me’ stimulated new areas of learning: (a) *a glimpse beneath the surface* explored a more pro-active way of seeing the person behind the pain (b) p*itfalls of the Medical Model* recognised the challenge of ‘sitting with’ rather than ‘fixing’; (c) *Feeling bombarded by despair* acknowledged the intense emotions that the clinicians brings to the clinical encounter; (d) *Reconstructing the clinical encounter as a shared journey* reconstructed the time-constrained clinical encounter as a single step in a shared journey towards healing.

People perform a picture of themselves to others [34] and performance is central to the experience of chronic pain [28]. Watching the film encouraged participants to recognise that appearances can be deceiving, and to see the value of delving beneath the surface. The film supports an embodied approach to healthcare that seeks the human aspects of patients’ experience [35]. Embodiment focuses on what is unique about this illness for *this* person. The theory of embodiment [36–41] is rooted in the thinking of Merleau-Ponty who breaks down the dualism of *mind and body* and focuses on integrating mind and body; we do not *have* a body, we *are* a body [35]. However, illness forces us to become aware of our body and thus creates a dualism that does not truly exist [36, 42].

Our findings suggest that qualitative films can stimulate a form of clinical knowledge that recognises the importance of the human story. It is through stories that people construct the their own sense of personal identity, and by listening to these stories we can understand people’s response to conditions that threatens this [6, 37, 43]. Self-Discrepancy Theory [44] incorporates three constructs related to personal identity that may underpin stories of illness: [1] A*ctual Self* - ‘your representation of the attributes that someone (yourself or another) believes you actually possess; [2] Ideal *Self* - ‘your representation of the attributes that someone (yourself *or another*) would like you, ideally, to possess’; [3] O*ught Self* - your representation of the attributes that someone (yourself or another) believes you should or ought to possess’. Discrepancies between actual, ideal and ought selves can lead to powerful emotions that we cannot understand without hearing a person’s stories. Watching and discussing the film encouraged participants to attend to potential threats to patients’ personal identity, and to invest clinical time to explore these losses with them.

The film demonstrated that the experience of, and response to, illness is the result of a complex relationship between biological and psychosocial factors [45]. There is a danger that psychosocial factors are ‘grafted onto’ biomedical care [46]. For example, participants suggested the possibility of dealing with the ‘psychological components’ separately. Embodied healthcare requires an understanding that ‘pathos precedes pathology’ ([39], pp. 137); life going wrong precedes a journey to the doctor. A truly embodied approach moves the clinician beyond an understanding of *seeing* as a physical act of vision, towards S*eeing* (with a capital S) that entails an effort to understand patients’ unique qualities. A modern metaphor for embodiment is the central theme in the 2009 film *Avatar, ‘I See you’.*

The power of the film hinges on making the person’s life ‘morally recognisable’ [43] and entails an ethical responsibility [31, 43]. Scarry argues that we are more likely to cause pain to people that we do not know*;* trying to ‘imagine other people better’ is therefore an important step towards an ethic that prevents us from (inadvertently or not) harming others [47]. This ethical responsibility can be an emotional experience for the healthcare professional and has a profound implication for clinical education – how do we equip clinicians with these skills? Watching the film ‘Struggling to be me’ encouraged participants to recognise and explore their own emotions. For example, it highlighted that treating patients with chronic, incurable conditions poses unique challenges: How do we balance an approach that embodies the individual with the ‘quiet despair’ that can accompany this; how do we empower clinicians to sit with, and yet not feel a sense of failure?

Medical Humanities focus on the added value of disciplines outside biomedicine for improved healthcare. For example, Charon demonstrates that s*itting alongside a patient*, although emotional, can have a positive impact on work satisfaction by facilitating clinical partnership and allowing clinicians to be generous with their presence [48]. Likewise, Levinas argues that seeing the ‘*Face’* of another can be a positive human experience for both parties; although *Seeing* the person can impose a burden, this ‘does not limit but promotes my freedom, by arousing my goodness’ ([49], pp. 200). Sitting alongside a patient frames the clinician as an advocate, rather than adversary. Intuitively, this is likely to have a positive effect on both healthcare professionals and their patients. Our findings support the use of films that portray qualitative research findings to facilitate a collaborative framework that is integral to a Shared Decision-Making Model.^2^

Our findings suggests that the construction of meaning from the film is firmly aligned with the themes described in the original systematic review [28, 29]. This indicates that the film did made viewers think about the themes as reported in the review. Our findings also support the view that *performing* qualitative research findings in film can *evoke*, *provoke* and *stimulate* ideas [13] by facilitating emotional engagement and empathy [2]. We did not explore whether or not reading a *written* report of the qualitative research would have a similar effect to watching it, and therefore we cannot be sure whether or not the impact of the film was due to the mode of dissemination or its content. It would therefore be useful to explore differences in impact from reading peer reviewed publications and other modes of dissemination. However, the film is short and easily accessible within an educational framework. We also do not know whether it was the *discussion* or the film per se that facilitated *knowing*. If we conceptualise *knowing* as a *dialectic,* process that occurs at the *interface* between research findings and audience [5, 7], it seems highly likely that discussing the film (either in the focus groups or individual interviews) played a role in co-constructing knowledge. As such, what we have captured is the construction of meaning, or *learning*, as developed within the interviews. We see this as strength of performative approaches that encourage participative engagement. More research is needed to research the differential impact of film, written report and discussion. However, our findings demonstrate that watching a ten minute qualitative film, followed by discussion, has a valuable educational potential. Although findings are context specific, we argue that findings are transferable across settings. It would be useful to know if the impact is the same for other groups such as undergraduate medical, nursing or allied health professional students.

## Conclusions

Our findings support the view that diverse forms of knowledge are relevant to clinical practice. This is a primary qualitative study exploring the impact on healthcare professionals of watching and discussing a short film that portrays the findings from a qualitative systematic review. Other audio-visual or commercial films could also contribute positively to clinical education. We demonstrate that the impact of qualitative research portrayed in film may be ‘a subtle shift in viewers perspectives’ [1] which encourages healthcare professionals (and others) to recognise the face of others. This *subtle shift* may be enough to inform more collaborative clinical encounters. Research-based qualitative films can make qualitative findings accessible and can be a useful resource in clinical training. Films that portray qualitative research findings can allow clinicians to recognise the emotions they bring into practice within a safe environment removed from the clinical encounter. Our research presents, for the first time, specific learning themes for clinical education. Importantly, in an educational setting, qualitative films can stimulate a pro-active and dialectic form of knowing, with implications for providing compassionate and person centred care.

## Footnotes

1 (https://www.youtube.com/watch?v=FPpu7dXJFRI)

2 (https://www.england.nhs.uk/ourwork/pe/sdm/).
